# Supplementary material for: The Small RNA Universe of Capitella teleta
Source: Front Mol Biosci. 2022 Feb 25;9:802814. doi: 10.3389/fmolb.2022.802814 (PMC8915122; doi:10.3389/fmolb.2022.802814)
Supplement: Supplementary file 1 [file DataSheet1.ZIP › Supplement/known/CAPTEscaffold_876_29816.pdf]

Provisional ID	:	CAPTEscaffold_876_29816
Score total	:	695.3
Score for star read(s)	:	3.9
Score for read counts	:	685.8
Score for mfe	:	0.9
Score for randfold	:	1.6
Score for cons. seed	:	3
Total read count	:	1357
Mature read count	:	1285
Loop read count	:	0
Star read count	:	72

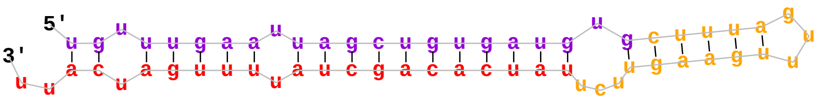

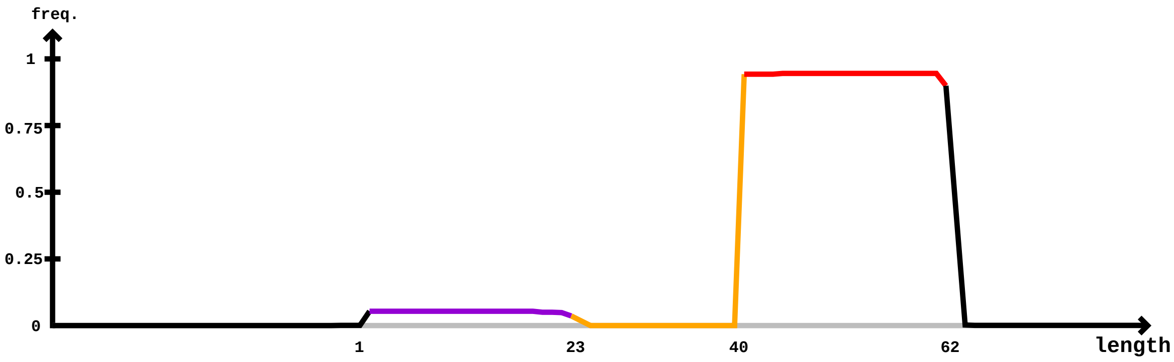

Star **Mature**

```
5'- guuuugucaugaguuucuagcuuugcucuaauguuugaauuagcugugaugugcuuuaguuugaaguucuuaucacagcuauuuugaucauuuuaacagaguuguuucguc   -3'   obs
    guuuugucaugaguuucuagcuuugcucuaauguuugaauuagcugugaugugcuuuaguuugaaguucuuaucacagcuauuuugaucauuuuaacagaguuguuucguc         exp
    guuuugucaugaguuucuagcuuugcucuaauguuugaauuagcugugaugugcuuuaguuugaaguucuuaucacagcuauuuugaucauuuuaacagaguuguuucguc         known
    ........(((((.....(((((((....((((.(((((.(((((((((((.((((((...))))))...))))))))))).))))).))))....)))))))...))))).   reads   mm   sample

    ............................uaauguuugaauuagcuguga..............................................................   1   0   seq
    ...............................uguuugaauuagcuguga..............................................................   4   0   seq
    ...............................uguuugaauuagcugugaug............................................................   2   0   seq
    ...............................uguuugaauuagcugugaugu...........................................................   16   0   seq
    ...............................uguuugaGuuagcugugaugu...........................................................   1   1   seq
    ...............................uguuugaauuagcugugaugug..........................................................   48   0   seq
    ...............................uguuugaauuagcugugaugugU.........................................................   1   1   seq
    ......................................................................uaucacagcuauuAugaucau....................   1   1   seq
    ......................................................................uaucacagcuauuuugaucau....................   62   0   seq
    ......................................................................uauAacagcuauuuugaucauu...................   1   1   seq
    ......................................................................uaucGcagcuauuuugaucauu...................   1   1   seq
    ......................................................................uaucacUgcuauuuugaucauu...................   1   1   seq
    ......................................................................uauUacagcuauuuugaucauu...................   1   1   seq
    ......................................................................uaucacagcuauuuugaucauG...................   1   1   seq
    ......................................................................uauGacagcuauuuugaucauu...................   1   1   seq
    ......................................................................Naucacagcuauuuugaucauu...................   1   1   seq
    ......................................................................Aaucacagcuauuuugaucauu...................   3   1   seq
    ......................................................................uaucacagcuauuAugaucauu...................   1   1   seq
    ......................................................................uaucacagcuauuuugauAauu...................   1   1   seq
    ......................................................................uaucacagcuauuuugaucauu...................   1192   0   seq
    ......................................................................uaucacagcuauuuugaAcauu...................   1   1   seq
    ......................................................................uaAcacagcuauuuugaucauu...................   1   1   seq
    ......................................................................uaucacagcuauuuAgaucauu...................   1   1   seq
    ......................................................................uaucacagcuauuuugaucaCu...................   1   1   seq
    ......................................................................uaucacagcuauAuugaucauu...................   1   1   seq
    ......................................................................uaucacagcuauuuugGucauu...................   1   1   seq
    ......................................................................uaucacagcuauuuuAaucauu...................   2   1   seq
    ......................................................................uaucacagAuauuuugaucauu...................   1   1   seq
    ......................................................................uaucacagcuauuuugaucauuA..................   3   1   seq
    ......................................................................uaucacagcuauuuugaucauuuA.................   2   1   seq
    ..........................................................................acagcuauuuugaucauu...................   4   0   seq
```

```
                                 Star                                     Mature
guuuugucaugaguuucuagcuuugcucuaauguuugaauuagcugugaugugcuuuaguuugaaguucuuaucacagcuauuuugaucauuuuaacagaguuuguuucguc
............................................................................................uuaacagaguuuguuucguc    1    0    seq
```
